# Response to the letter to the Editor “Comparing video and direct laryngoscopy for tracheal intubation in the general ward” by Shao and Colleagues

**DOI:** 10.1186/s13613-019-0510-2

**Published:** 2019-03-07

**Authors:** Moon Seong Baek, MyongJa Han, Jin Won Huh, Chae-Man Lim, Younsuck Koh, Sang-Bum Hong

**Affiliations:** 1 Department of Pulmonary and Critical Care Medicine, Asan Medical Centre, University of Ulsan College of Medicine, 88 Olympic-ro 43-gil, Songpa-gu, Seoul, 05505 Republic of Korea; 2 Medical Emergency Team, Asan Medical Centre, University of Ulsan College of Medicine, Seoul, Republic of Korea

## Reply

This letter is in response to the letter by Shao and colleagues. We thank for their interest in our work and comments.

First, the medical emergency team of Asan Medical Center has consistently provided a tracheal intubation procedure. The sniffing position has pursued to achieve the best laryngeal exposure for direct laryngoscopy. A doughnut-shaped pillow was placed under the patient’s occiput to elevate head, and then, neck flexion with upper cervical extension was obtained [1]. Although the video laryngoscopy did not require the sniffing position, video laryngoscopy group was placed in the sniffing position if possible. Therefore, we suggest that the positioning is not critical factor to influence the outcomes.

Second, difficult airway prediction based on LEMON score can be useful in the emergency setting. However, LEMON has never been validated in out-of-operating room, and MACOCHA score is the only validated score predicting the difficulty of intubation procedure [2]. LEMON score could not be presented in our study. The patient may be falsely judged to be difficult to intubate if each single factor is considered as difficult airway. However, serious consequences in the airway management can be resulted by unanticipated difficult airway rather than false positive prediction of difficult airway [3]. Besides, 20% of overall rate of difficult airway is comparable to previous studies.

Third, our study results showed median 4 min of intubation time. As we described in methods section, we defined intubation duration as the time between infusion of the “pre-treatment agent” and confirmation of tracheal tube placement by capnography. Induction drug was administered after 2 min of pre-treatment agent injection. Generally, intubation duration is defined as the time from the administration of “induction drugs” to the confirmation of tube placement in the trachea, and the median duration was 3 min in the MACMAN trial [4]. Therefore, overall intubation duration seems to be similar compared to the other trial.

Fourth, we entirely agree with the opinion of Shao et al. that experienced intubators in this study could not accurately indicate the competency levels of intubators with video laryngoscopy and direct laryngoscopy. However, in subgroup analysis of our study, inexperienced residents in training have significantly higher success rate of intubation in video laryngoscopy group than in direct laryngoscopy group (75% vs. 52%, *p* < 0.001) (Table 7). Besides in experienced doctors, the success rate was not different, but higher tendency between video laryngoscopy and direct laryngoscopy (86.4% vs. 78.5%, *p* = 0.068).

For all these reasons, despite the comments raised by Shao and colleagues, we think that our work had a good internal and external validity to assess the real efficiency of video laryngoscopy and direct laryngoscopy for urgent intubation in the general ward.
